# Circulating miR-206 as a Biomarker for Patients Affected by Severe Limb Girdle Muscle Dystrophies

**DOI:** 10.3390/genes12010085

**Published:** 2021-01-12

**Authors:** Valentina Pegoraro, Corrado Angelini

**Affiliations:** IRCCS San Camillo Hospital, Via Alberoni 70, 30126 Venice, Italy; valentina.pegoraro@ospedalesancamillo.net

**Keywords:** LGMD, miR-206, myomiRNAs, biomarkers, transportinopathy, sarcoglycanopathy, calpainopathy, muscle MRI

## Abstract

Limb-girdle muscular dystrophies (LGMD) are clinically and genetically heterogeneous conditions, presenting with a wide clinical spectrum, leading to progressive proximal weakness caused by loss of muscle fibers. MiR-206 is a member of myomiRNAs, a group of miRNAs with important function in skeletal muscle. Our aim is to determine the value of miR-206 in detecting muscle disease evolution in patients affected by LGMD. We describe clinical features, disease history and progression of eleven patients affected by various types of LGMD: transportinopathy, sarcoglycanopathy and calpainopathy. We analyzed the patients’ mutations and we studied the circulating miR-206 in serum by qRT-PCR; muscle MRI was done with a 1.5 Tesla apparatus. The severe evolution of disease type is associated with the expression levels of miR-206, which was significantly elevated in our LGMD patient cohort in comparison with a control group. In particular, we observed an over-expression of miR-206 that was 50–80 folds elevated in two patients with a severe and early disease course in the transportinopathy and calpainopathy sub-types. The functional impairment was observed clinically and muscle loss and atrophy documented by muscle MRI. This study provides the first evidence that miR-206 is associated with phenotypic expression and it could be used as a prognostic indicator of LGMD disease progression.

## 1. Introduction

The limb-girdle muscular dystrophies (LGMD) include a group of clinically and genetically heterogeneous diseases that lead to progressive weakness, predominantly proximal to the presentation, caused by loss of muscle fibers. The new revised classification of LGMDs, proposed during the 229th International European Neuro Muscular Centre (ENMC) Workshop [1], is based on genetic inheritance, dominant (D) or recessive (R) and the order of discovery (number) of the gene/protein concerned, and on clinical, MRI, histopathological features. Common clinical signs useful for classification include that affected individuals must achieve independent walking ability and laboratory-based data, such as elevated (CK)and muscle biopsy histopathology showing dystrophic features, eventually leading to end-stage disease and observation of degenerative changes in MRI [1,2,3]. LGMDs are characterized by a variable clinical course with progressive weakness with onset in the proximal girdle muscles, with the age of onset of symptoms ranging from early childhood (non-congenital) to late adulthood [3,4]. The progression of muscle weakness is usually symmetrical and variable between individuals and the genetic type. These disorders present a broad spectrum of muscle involvement and atrophy, ranging from severe with early-onset and rapid progression, such as those with childhood-onset, to a milder form with late-onset.

In our cohort we document changes in the transportinopathy LGMD-D2, a rare dominant form of LGMD due to a mutation in *TNPO3* gene, encoding for transportin-3 protein, a nuclear import receptor for serine/arginine-rich proteins which is important for mRNA splicing [5,6].

This form was identified for the first time in a large Italo-Spanish family [7], in which the major clinical features are weakness in the proximal limb-girdle muscle and variable onset. The main clinical signs in LGMD-D2 are severe weakness evident first in the pelvic girdle muscle and then in the shoulder girdle, while the severe cases were characterized also by distal muscle weakness, scoliosis, winging scapulae and occasionally by facial weakness [8,9]. Cognitive impairment or cardiac involvement has not been described in LGMD-D2, while respiratory or bulbar involvement has been observed only in early-onset cases presenting severe phenotype [7,8].

Another type of LGMD that we will consider are the group of sarcoglycanopathies caused by mutations in the genes that code for four muscle-specific proteins called α-, β-, γ- and δ-sarcoglycan, subunits of the sarcoglycan complex associated with dystrophin in cardiac and skeletal muscle [10,11]. Sarcoglycans form a complex essential for muscle membrane stability during contraction, as they are crucial components of the dystrophin-glycoprotein complex that physically connects the intracellular cytoskeleton to the extracellular matrix [12]. Mutations in one of the sarcoglycan genes can cause the loss of this structural bond, making muscle fibers more susceptible to damage during muscle contractions [11].

The phenotypes of sarcoglycanopathies are similar to those of dystrophinopathies, except for the absence of cognitive dysfunctions [13] and for the appearance of winging scapulae. The most common childhood types of α, β and γ sarcoglycanopathy is manifested by a clinical phenotype similar to that observed for Duchenne muscular dystrophy, with onset of weakness in childhood and the disease is more severe and rapid than in other LGMDs. Patients with defects in sarcoglycan genes might present cardiac involvement (manifesting as dilated cardiomyopathy) often compared to other forms of LGMD [10,14]. Early childhood onset is common but onset in adults is due to α, β γ sarcoglycan defects. There is presence of Gowers sign and inability to run and jump, sports ability can be affected in childhood or preserved up to middle age [13], muscle hypertrophy is present in the calves and tongue.

We also describe a group of people affected by calpainopathy (LGMD-R1), the most frequent form of girdle dystrophy, which in European countries would represent about 40–50% of total cases of LGMD [15,16,17,18,19], a recessive form, due to mutations in the gene coding for calpain-3 (*CAPN3*), a muscle specific family’s member of Ca^++^ activated neutral protease [20]. The identification of the disease locus in chromosome 15 [21], the first mutations in the *CAPN3* gene were then identified both in France [22] and clinically described in a group of LGMD-R1 patients who lived in a small community [23]. The discovery of this isolate was followed by the identification of other LGMD-R1 genetic clusters including in other specific environments (e.g., in the Alps [24] or in the Venetian lagoon [25]).

Clinically LGMD-R1 is characterized by selective and progressive involvement of the proximal limb-girdle muscles. Based on the distribution of muscle weakness at the onset, two main forms have been identified: Leiden-Möbius pelvic-femoral, which is the most frequently observed, in which muscle weakness occurs first in the pelvic girdle and then in the shoulder girdle; and the scapulohumeral form of Erb, which is usually a milder phenotype, rarely early onset, in which muscle weakness is first evident in the shoulder girdle and only later in the pelvic girdle [26]. Levels of CK are consistently high since childhood (5–80 times normal). The age of onset of muscle weakness varies between two and 40 years (average 15 years). The first clinical symptoms to appear are generally difficulty in running, a tendency to walk on tiptoes and scapular wing caused by weakness of the shoulder girdle muscles. Weakness and wasting of the hip adductor/extensor and hamstring muscles (gluteus maximus, semimembranous, hamstring) can be seen both on clinical examination and on CT and muscle MRI. Muscle contractures may also be evident early. As the disease progresses, waddling gait, difficulty climbing stairs, lifting weights, and getting up from the floor or chair may be noted. Muscle involvement is mainly symmetrical and atrophic while the face and neck muscles are usually spared.

No efficient therapies are available for LGMD; CK has often been used as biomarkers but this is influenced by the patient’s clinical status and drug therapy; therefore, the discovery of new non-invasive biomarkers is needed.

MyomiRNAs are a group of highly expressed miRNAs in skeletal muscle. The myomiRNAs family includes miR-1, miR-133a, miR-133b and miR-206, which perform important functions in muscle and are used as easily available biomarkers in various neuromuscular diseases [27,28].

In this study, we describe the clinical features, mutations, disease history and progression of eleven LGMD patients. Circulating miR-206, with an important role in muscle recovery and regeneration, is evaluated in our cohort as a good candidate for a circulating biomarker of prognosis and we evaluate possible disease progression associated with muscle MRI imaging to monitor muscle damage.

## 2. Materials and Methods

### 2.1. LGMD Patients and Clinical Examination

In this study, we describe eleven patients affected by LGMD (aged 12–65 years), eight female and three male with a clinical phenotype of transportinopathy, calpainopathy and sarcoglycanopathy. The genetic gene mutations of transportinopathy and calpainopathy cases were analyzed on genomic blood DNA in collaboration with TIGEM (Telethon Institute of Genetics and Medicine) or other genetic units. The muscle biopsy was previously done in sarcoglycanopathy cases at University of Padua. Our patients present no contraindications to performing muscle MRI. Seven healthy volunteers matched for sex and age (range age 18–61 years) were used as control group.

Most patients underwent periodical clinical examinations, the first at the time of diagnosis and then during follow-ups as the disease progressed. Concurrently with the last medical neurological examination at neuromuscular Center, Neurobiology Lab, of IRCCS San Camillo (Venice, Italy) the patients underwent to muscle MRI and blood collection for miRNAs analysis. In this occasion, all patients or their parents, in case of subjects under the age of 18 years old, and healthy individuals signed the informed consent for the collection of biological samples in Biobank BBMRNR (Venice, Italy), for muscle MRI and for clinical data recording. During the visit muscle condition, MRC (Medical Research Council) score, gait scoliosis, scapular winging, difficult in climbing stairs or performing Gowers’ maneuver, age and onset of first symptoms were recorded. In this study, we considered: Early-onset < 10 years, early adolescence onset 10–14 years, late-onset 20–30 years. The functional severity score (FSS) of disease evolution at last examination was graded as follows: Score 3 indicates severe and rapid evolution worsening of symptoms over the past 2 years; score 2 indicates moderate and progressive evolution; score 1 mild with a long history of illness. The approval from the local ethics committee was obtained for this study.

### 2.2. MicroRNAs Analysis

Total RNA was extracted from 400 μL of serum samples with the miRNeasy Mini Kit (Qiagen, Hilden, Germany), as recommended by the manufacturer. After the addition of QIAzol Lysis reagent and the incubation with chloroform, 10 µL of 5 nM of miR-39-3p of *C. elegans* was added, as previously described [29].

Quantification of serum miR-206 in each patient and control subjects was made by qRT-PCR with a two-step process: First 5 µL of total RNA was reverse transcribed using TaqMan miRNAs Reverse Transcription Kit (Applied Biosystems, Carlsbad, CA, USA). In the second step, cDNA was amplified by Real-Time PCR using the CFX96™ RT-PCR Detection System (Biorad, Hercules, CA, USA) (Bio-Rad), along with specific TaqMan MicroRNA Assay (Thermo Fisher Scientific, Rodano, Italy). The normalization was made to the average of miR-16, U6 snRNA, and miR-39-3p of *Caenorhabditis* as previously described [30,31]. The relative expression of miR-206 was calculated using the ΔΔCt method.

### 2.3. Muscle MRI

Patients underwent skeletal muscle lower limbs MRI using 1.5-Tesla Philips Apparatus with T1-weighted MRI system. The fibrofatty replacement of muscle was evaluated using Mercuri score [32].

### 2.4. Statistical Analysis

Statistical analysis was performed using R-Studio program. The Wilcoxon-Mann-Whitney test was used for small samples for miRNA’s analysis. Spearman rank test was used to correlate the age at last examination with the functional severity score. Statistical significance was *p*-value of ≤0.05.

## 3. Results

### 3.1. LGMD Clinical and Genetic Features

We studied eleven LGMD patients from nine families: Two couples of mother and child, two patients had a positive family history and the rest were isolated cases. At the time of the study, three patients had lost ambulation. Clinical features and molecular data are reported in Table 1. The type of mutation and domain protein involved has been included.

**Figure 1:**
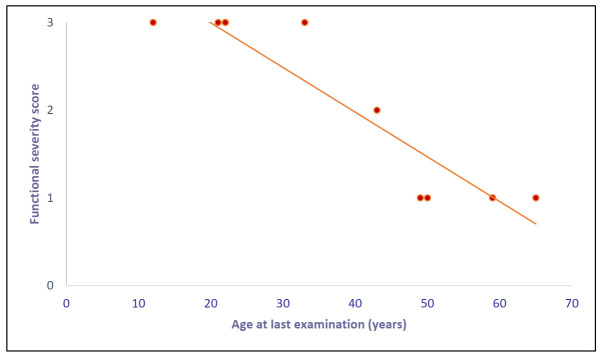
Correlation between “age at last examination” and “functional severity score”: We found a significant (Spearman rank test) inverse correlation. The graph shows nine points because two couples of patients (D-G and E-L) have the same age and score and so the points are overlapping.

We explored the relationship between age at last examination and functional severity score: We observed a significant inverse correlation—the younger patients presented also a more rapid and severe disease evolution (Figure 1).

Our patients are subdivided into three groups (transportinopathy, calpainopathy and sarcoglycanopathy) according to gene mutations. MRC and Mercuri score are reported in Table 2 and Table 3.

#### 3.1.1. Transportinopathy

This group consists of two duos of mother and child, respectively, of Hungarian and Italo-Spanish origin, with LGMD-D2 myopathy phenotype, respectively, due to two different single nucleotide deletions in the *TNPO3* gene.

Patient A, a 12-year-old boy, presented a congenital myopathy phenotype from the first years with difficulty raising from the floor. At the last examination he reported a rapid and severe worsening, in a short time he went from walking a couple of km in the mountains to walking only 50–100 m on level ground with the help of the rail. His 43 year-old mother (patient B) showed a moderate and progressive evolution of the limb-girdle disease.

In the second family, the daughter (patient C) of 33 years old and her mother (patient D) of 59 years old showed atrophy of upper girdle muscles, especially in trunk, deltoid and triceps, difficulty in lifting arms over the head, arachnodactyly and scoliosis. The daughter’s muscle MRI shows advanced atrophy and fat/connective substitution in gluteus minimus and medius, of posterior thigh muscle semitendinosus and anterior thigh vastus lateralis, sartorius and tensor fasciae latae. There is atrophy of tibialis posterior, flexor hallucis longus and gastrocnemius. The mother is doing relatively well, although her muscle biopsy shows more advanced signs of muscle fiber splitting and segmentation. The muscle MRI shows atrophy of glutei and posterior thigh muscles. There is also atrophy of anterior thigh compartment particularly in vastus lateralis and rectus femoralis and advanced connective substitution of sartorius, tensor fasciae latae and both gastrocnemius muscles.

#### 3.1.2. Calpainopathy

This group is composed by three non-consanguineous cases and one consanguineous case.

Patient E is a 22-year-old man with a severe and rapid evolution of the disease. His parents came from a small village in Moldavia. He had an early onset progressive weakness of lower limb and was not able to run or walk fast, has difficulty climbing stairs that he can do only with the help of handrail and is not able to perform Gowers’ maneuver. Muscle strength graded by MRC scale is 4/5 in biceps brachialis and quadriceps, and 3+/5 in iliopsoas. His biceps muscle is atrophic, as well as calf muscles, and has bilateral pes cavus. His CK is 7060 U (n.v. <308 U) and the EMG was myopathic.

He is in therapy with methylprednisone 16 mg/day and vitamin D with no benefit.

Muscle MRI shows marked atrophy with adipose involution of the posterior glutei and thigh muscular structures, and minor atrophy of the posterior components in the calf.

Patient F is a female of 50 years old. At the last visit, the patient has bilaterally scapular winging, slightly waddling walk, scoliosis and she complains of pain in the back leg. One sister and her daughter have elevated CK. In her muscle biopsy a myopathy was observed: Where was type 1 fiber predominance with atrophic fibers of both types.

Her muscle MRI shows marked fibro adipose substitution on gluteus and bilaterally of posterior thighs muscles. She was carrying a heterozygous missense mutation in the *CAPN3* gene (1486 G > A in exon 11, with amino acid substitution p.G496R).

Patient G: This 59-year-old woman has been suffering from lower limb weakness since age 17. EMG showed myopathic sign and a muscle biopsy was done were inflammatory signs with macrophagic infiltration was observed, for which she was seen by Lord Walton at age 18, and she was found to suffer from a LGMD myopathy. She has used a wheelchair since age 45. Her CK was 1025 U (n.v. up to 50 U). She has suffered from hypertension since 50 years of age.

She carries two heterozygous mutation of the *CAPN3* gene.

Patient H, a 49-year-old patient, was born from a consanguineous family affected by weakness and fatigability, for which, at 23 years old, a muscle biopsy was done.

The weakness was mostly in lower girdle; he had a wadding gait, winging scapulae and was diagnosed as affected by LGMD. He was treated with steroids and BCAA infusion. A mutation of *CAPN3* gene was found in exon 21 homozygous. He has been WCB (wheelchair bound) since age 49.

#### 3.1.3. Sarcoglycanopathy

This group is composed by three female patients that presented a mutation in γ (patient I), β (patient L) and α (patient M) sarcoglycan gene, respectively.

Patient I is a 21-year-old female of Tunisian origin. The onset of the disease was at about 12 years old when the patient began to notice easy fatigability; she was no longer able to sustain the running competitions with her peers. Subsequently, a waddling gait on tiptoes and proximal weakness of the lower limbs appeared. In 2009, she performed muscle biopsy that showed marked fibers variability with numerous hypertrophic and atrophic fibers, splitting and centralized nuclei. Recent MRC scores are present in Table 2.

Patient L, a 22-year-old female, diagnosed at three years of age with elevated CK value (22,206 U), showed a progressive muscular dystrophy and dystrophic muscle biopsy. At three years old she had scapular winging, Gowers’ sign and was unable to climb stairs. At six years of age, a muscle CT scan showed no significant atrophy. In this patient, a muscle MRI was not performed. She became wheelchair bound at age 12 years. During the following years, she developed cardiac involvement with reduction of ejection fraction, diffuse ventricular hypokinesia and life threatening arrhythmias for which she was in list for cardiac transplantation.

Patient M. This 65-year-old woman had lower-girdle weakness for which, at age 31. she was hospitalized and a biopsy was done. She had in her history a consanguineous family with a diagnosis of LGMD entertained. She had at α sarcoglycan immunohistochemistry a partial reduction of staining.

She was found to carry a homozygous change in α-sarcoglycan gene C > T at nucleotide 850. Muscle MRI was not performed.

### 3.2. miR-206 in LGMD Patients

In this study, we carried out the analysis of circulating miR-206 expression levels in serum of eleven patients with LGMD: four transportinopathy, four calpainopathy and three sarcoglycanopathy in comparison with a healthy group.

**Figure 2:**
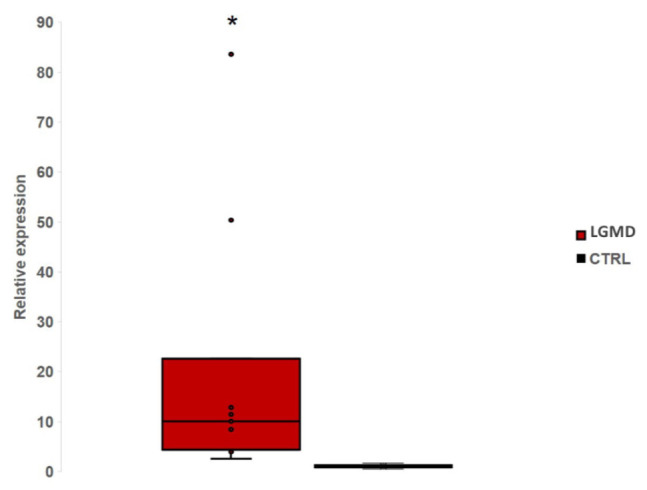
The Box-plot shows the relative expression level of miR-206 in eleven LGMD patients comparing with controls. We found a significant (*p* < 0.05) up-regulation of miR-206 in LGMD patients. *: significant value.

MiR-206 was found significant (*p* < 0.05) dysregulated in our whole group of patients compared with healthy controls (Figure 2).

**Figure 3:**
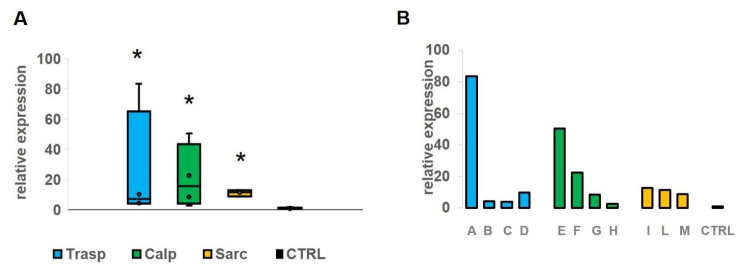
(**A**) The figure shows the expression level of miR-206 in four patients with transportinopathy (blue), in four with calpainopathy (green) and in three with sarcoglycanopathy (yellow). We found a significant (*p* < 0.05) up-regulation of miR-206 in all groups comparing with the control group. (**B**) On the right (panel B) we show a single patient value of miR-206 that appears particularly elevated in patients A and E in comparison with the mean of controls. *: significant value.

To assess whether there was a difference in miR-206 expression level between the clinical subtypes of the disease, we divided the sample into three subgroups (transportinopathy in blue, calpainopathy in green and sarcoglycanopathy in yellow). We found a significant up-regulation (*p* < 0.05) of miR-206 in all groups compared to controls (Figure 3A). The expression level of miR-206 appears with a very variable range in the first two groups, from about four to 80 folds in patients with transportinopathy and three to 50 folds in patients with calpainopathy, while in the sarcoglycan group we observed elevation of approximately 11 folds on average. Of particular note, we found a 50–80-fold overexpression of miR-206 levels in two patients (Figure 3B, Patients A and E), with a severe and very rapid disease course compared to controls.

## 4. Discussion

The study of micro-RNA in limb-girdle patients are lacking. particularly the one on myomiRNAs, which are a group of highly expressed miRNAs in skeletal muscle. The myomiRNAs family includes miR-1, miR-133a, miR-133b and miR-206, with important functions in muscle homeostasis, regeneration and development [27,33,34]. Circulating myomiRNAs release in the bloodstream by muscles in response to physiological or pathological muscle processes, which could act as cell signaling mediating cell-to-cell communication regarding muscle repair, regeneration and remodeling [35,36]. The mechanisms by which miR-206 enter in the blood circulation is two-fold: On one side there is an active secretion of microvesicles from muscle tissue to capillaries, on the other hand the breakdown of muscle fiber due to dystrophic process contributes to release of miR-206 in blood.

The potential role of circulating myomiRNAs as easily available biomarkers have begun to gain great interest and changes in miRNA expression level have been described in several muscle diseases [27]. Matsuzaka et al. [37] observed elevated levels of miR-206 in serum of Becker muscular dystrophy (BMD) patients, with a 2.1-fold increase in respect to control, while miR-1 levels in the serum of patients with LGMD, FSHD (Facio-Scapilo-Humeral Dystrophy) and BMD were, respectively, 5.5, 3.3 and 1.7 relative to control sera. There were seven LGMD cases separated by a LGMD subgroup; however, after Bonferroni correction no significant differences were found and; therefore, an increase in sample was needed to evaluate these three biomarkers. In another study [38], a total of 187 miRNAs were investigated in 15 muscle biopsies from 10 calpainopathy and five dysferlinopathy patients: A cluster of dysregulated miRNAs were found to be involved primarily in inflammation and calcium metabolism. They showed opposite regulation in calpainopathy versus dysferlinopathy cases, possibly in relation to different metabolic pathways identified by bioinformatics and morphologic approach, suggesting a high degree of activation of intracellular regulation of calcium in dysferlinopathy, that is characterized by high CK and often with inflammation in muscle biopsy.

Israeli et al. [39] observed that myomiRNAs dysregulation was first observed in muscle biopsy and later extended to plasma samples, suggesting that this spill-over might be of interest for their role as biomarkers. In a mouse model of α-sarcoglycanopathy there was during gene therapy a dose-response correlation and such dose-response was found on the expression of miRNAs both in muscle tissue and in blood, this study supports the utility of profiling circulating miRNAs to study and monitor the evolution of muscle diseases. It is important the appropriate choice of biomarkers to monitoring, evaluate and investigate the evolution of disorders with clinical heterogeneity, such as LGMD or BMD.

In this study, we describe the clinical features, disease history and progression of eleven genetically-defined LGMD patients, including the new group of the dominant LGMD-D2 transportinopathy. We analyzed the patients’ mutations and studied the circulating serum miR-206; muscle impairment was assessed by muscle MRI. This study documents that the higher level of expression was found in transportinopathy, particularly in a young patient that was having a fast evolution and in a few years was losing independent ambulation. A similarly high level was found in a young 21-year-old calpainopathy patient that similarly had difficulty walking, climbing stairs and progressed fast in the following years. Additionally, the other cases analyzed in this study presented significant increased levels of miR-206 in comparison with a control group. MyomiRNAs were previously studied by Matsuzaka [37] and by our group [40] in LGMD and Becker with variable levels of elevation in patients, suggesting that perturbation of muscle metabolism in muscular dystrophy patients is variable and linked to disease evolution. In another study [41], dystromiRs were found upregulated in Duchenne Muscular Dystrophy (DMD): Patients with moderate disease presented higher levels of miR-206 than more severely affected and they proposed miRNAs as valid biomarkers to follow the progression disease. Cacchiarelli in DMD [42] has found high levels of miR-206, possibly indicating a high level of muscle degeneration and regeneration.

We hypothesized that high levels of miR-206 reflect a regenerative process in patients with active LGMD evolutive disease. Of particular interest, in vitro evidence showed that an up-regulation of miR-206 occurs during skeletal muscle regeneration by restricting proliferative potential and promoting SCs (Stem cells) differentiation [43,44]. Otherwise, in mdx model mice, it was seen that lack of miR-206 accelerates muscle dysfunction in response to cardiotoxin injury, with delay of regeneration due to inefficient SCs differentiation [33]. On the other hand, the severity of LGMD, due to decrease in muscle mass, is a consequence of evolution of fibrosis, as documented in our patients by muscle MRI. We speculated that this leads to a reducing level of miR-206 in patients with major fibrosis due to a small number of muscle cells in comparison with other patients.

No efficacy therapies are available for LGMD, but, only in few α-sarcoglycanopathy cases, steroids influenced the patient’s clinical status with drug therapy, while in the β-sarcoglycanopathy patient we report a subsequent heart transplant was done with success, since his skeletal muscle weakness was not deteriorating.

A limitation of this study is that we do not have a serum/plasma sample after cardiac transplant and, in general, longitudinal studies would be desirable in our LGMD cases. Such studies could also be correlated to other imaging or circulating biomarkers and clarify if changes in levels could be attributed to a regeneration process in LGMD, or reflect late stage of disease with increased fiber autophagy, necrobiosis and connective/fat substitution.

We need further validated observations, including the longitudinal study in groups of evolving cases, as well as an additional cohort of LGMD cases to analyze changes in a series with wide age distribution.

## 5. Conclusions

We document a variable expression of miR-206 up-regulation in eleven LGMD patients, which was particularly evident in two cases with a rapid severe clinical evolution. As to whether this might reflect an important role in muscle recovery and regeneration, we observed a correlation of outcome measures in relation to the evolution and change of this biomarker. Our study supports the emerging role of miR-206 as a good candidate as an easily available circulating biomarker of prognosis, and in monitoring the progression of muscle damage and remaining muscle mass associated with muscle MRI imaging in muscular dystrophy.

## Figures and Tables

**Table 1:** Clinical and molecular features in eleven Limb-Girdle muscular dystrophy (LGMD) patients.

Clinical and Molecular Features in Eleven LGMD Patients									
pt.	Sex	Age at Last Examination	Onset	FSS	LGMD Subtype	Gene	Chromosome	cDNA Variant	Protein Amino-Acid Change
A	M	12	early	3	LGMDD2	*TNPO3*	7q32.1-q32.2	Frameshift mutation c.2767delC (exon 23)	p.Arg923AspfsTer17
B	F	43	early	2	LGMDD2	*TNPO3*	7q32.1-q32.2	Frameshift mutation c.2767delC (exon 23)	p.Arg923AspfsTer17
C	F	33	early	3	LGMDD2	*TNPO3*	7q32.1	Frameshift mutation c.2771delA (exon 22)	p.X924Cys
D	F	59	late	1	LGMDD2	*TNPO3*	7q32.1	Frameshift mutation c.2771delA (exon 22)	p.X924Cys
E	M	22	early adolescence	3	LGMDR1	*CAPN3*	15q15.1-q21.1	c.550delA (exon 4) Missense mutation c.1657G˃A (exon 13)	p.Thr184ArgfsTer36 p.Glu553Lys
F	F	50	late	1	LGMDR1	*CAPN3*	15q15.1-q21.1	Missense mutation c.1486G˃A (exon 11)	p.Gly496Arg
G	F	59	early	1	LGMDR1	*CAPN3*	15q15.1	Missense mutations c.202T˃C (exon 1) c.1061T˃C (exon 8)	p.Cys68Arg p.Val354Gly
H	M	49	late	1	LGMDR1	*CAPN3*	15q15.1	Missense mutation c.2242C˃G homozygous (exon 21)	p.Arg748Gly
I	F	21	early	3	LGMDR5	*SGCG*	13q12.12	c.525delT homozygous	p.Phe175Leufs
L	F	22	early	3	LGMDR4	*SGCB*	4q12	Nonsense mutation c.594T˃G c.418-425dup8bp	p.Gln142Lysfs24Ter
M	F	65	early adolescence	1	LGMDR3	*SGCA*	17q21.33	Missense mutation c.850C˃T homozygous	p.Arg284Cys

Legend: pt: patient. FSS: Functional severity score of disease evolution: 3—severe and rapid evolution; 2—moderate and progressive evolution; 1—mild and long disease history. Early onset < 10 years; early adolescence onset 10–14 years; late onset 20–30 years.

**Table 2:** MRC scale.

MRC SCALE				
PATIENTS	QUADRICEPS	HAMSTRINGS	TIBIALIS ANTERIOR	GASTROCNEMIUS
A	3/5sx 3/5dx	3/5sx 3/5dx	4/5sx 4/5dx	4/5sx 4/5dx
B	3/5sx 3/5dx	4/5sx 4/5dx	3/5sx 3/5dx	2/5sx 2/5dx
C	4/5sx 4/5dx	3/5sx 4/5dx	3/5sx 4/5dx	2/5sx 2/5dx
D	4/5sx 4/5dx	4/5sx 4/5dx	4/5sx 4/5dx	4/5sx 4/5dx
E	4/5sx 4/5dx	n.a.	4/5sx 4/5dx	4/5sx 4/5dx
F	5/5sx 5/5dx	3/5sx 3/5dx	5/5sx 5/5dx	5/5sx 5/5dx
G	2/5sx 2/5dx	2/5sx 2/5dx	2/5sx 2/5dx	2/5sx 2/5dx
H	2/5sx 2/5dx	2/5sx 2/5dx	2/5sx 2/5dx	2/5sx 2/5dx
I	3+/5sx 3+/5dx	3+/5sx 3+/5dx	5/5 sx 4+/5 dx	5/5sx 5/5dx

Legend: MRC: Medical Research Council; sx—left, dx—right; n.a.: data not available; data from patients L and M are not available.

**Table 3:** Mercuri score in muscle of Limb-Girdle muscular dystrophy.

Mercuri Score in Muscle of Lgmd Patients																										
	Thigh																Leg									
Patient	Rectus Femoris		Vastus Lateralis		Vastus Medialis		Sartorius		Biceps Femoris		Semimenbranosus/Semitendinosus		Adductor Magnus		Gracilis		Anterior Tibial		Extensor Longi Digitorum and Hallucis		Peroneus		Gastrocnemius		Soleus	
	dx	sx	dx	sx	dx	sx	dx	sx	dx	sx	dx	sx	dx	sx	dx	sx	dx	sx	dx	sx	dx	sx	dx	sx	dx	sx
A	3	3	3	3	3	3	3	3	4	4	2a	2a	4	4	2a	2a	2a	2a	2a	2a	2a	2a	2b	2b	2a	2a
B	4	4	4	4	4	4	4	4	4	4	2b	2b	4	4	3	3	2b	2b	2b	2b	2b	2b	4	4	3	3
C	2b	2b	2b	2b	2a	2a	3	3	2a	2a	2b	2b	2b	2b	3	3	2a	2b	2a	2b	2a	2b	4	4	2a	2a
D	3	4	2b	2b	2b	2b	4	4	2b	2b	2b	2b	2b	2b	3	3	2b	2b	2b	2b	2b	2b	4	4	2a	2a
E	3	3	3	3	3	3	3	3	4	4	3	3	4	4	3	3	2a	2a	2a	2a	2b	2b	3	3	2a	2a
F	0	0	1	1	1	1	0	0	2b	2b	3	3	3	3	0	0	0	0	0	0	1	1	1	1	0	0
G	3	3	3	3	3	3	3	3	4	4	4	4	4	4	2b	2b	2a	2b	2a	2a	2b	2b	4	4	4	4
H	3	3	3	3	3	3	3	3	4	4	4	4	4	4	3	3	/	/	/	/	/	/	/	/	/	/
I	2a	2b	2b	2b	3	3	2a	2a	3	3	4	4	1	1	1	1	2b	2a	2b	2b	2b	2b	1	1	2a	2b

Legend: sx—left, dx—right; /—not available; patients L and M did not perform muscle MRI.

## Data Availability

Not applicable. All patient data is provided in this article anonymously.
